# Mapping urban well-being with Quality Of Life Index (QOLI) at the fine-scale of grid data

**DOI:** 10.1038/s41598-024-60241-0

**Published:** 2024-04-27

**Authors:** Ewa Dobrowolska, Katarzyna Kopczewska

**Affiliations:** Faculty of Economic Sciences, University of Warsaw, Ul. Dluga 44/50, 00-241 Warsaw, Poland

**Keywords:** Quality of life, Well-being, Urban economics, Urban planning, Proximity, 15-min city, Environmental social sciences, Environmental economics, Sustainability

## Abstract

Accessibility of transport infrastructure, commercial amenities, recreational facilities, and green spaces is widely recognised as crucial to the well-being of urban residents. However, these features are often unevenly distributed across the geographical boundaries of a city, leading to disparities in the local quality of life. This study focuses on the city of Warsaw, Poland, and uses the aforementioned characteristics and the framework of the '15-min city' concept to construct a grid-level urban Quality of Life Index (QOLI) that facilitates comparisons between the city’s districts and local neighbourhoods. The results of our study reveal a “high-inside, low-outside” pattern of quality of life, characterised by higher standards of living in the central districts and lower standards at the city's periphery.

## Introduction

Quality of life (QOL) is one of the key concepts for successful urban planning and is therefore often addressed in planning policies worldwide. For example, the European Union’s main objective is to “promote the well-being of its citizens”^1^, which is why data on subjective satisfaction with life in the EU is collected and analysed every year. The OECD’s “How's Life?” programme^2^ uses more than 80 indicators and shows improvements in life satisfaction as well as persistent inequalities. Countries, regions and cities compete with each other to attract inhabitants and businesses. Offering an exceptional standard of living is of primary importance.

The concept of quality of life is multidisciplinary and multidimensional. It is of interest not only to urban planners but also to sociologists, psychologists and economists. For example, planners develop urban geo-design to facilitate social interactions between the city residents; internal mechanisms of life satisfaction and evaluation of the perceived environment is a psychological matter. Economists, on the other hand, see environmental features and quality of life as economic goods that stimulate inter- and intra-urban migration, entrepreneurship and urban growth. Because of its complex nature, quality of life is difficult to define precisely. The World Health Organization defines QOL as “an individual’s perception of their position in life in the context of the culture and value systems in which they live and in relation to their goals, expectations, standards and concerns”^3^; However, some studies simply associate it with general life satisfaction or well-being^4^. In fact, more than 100 definitions have been presented in the literature so far^5^. In this paper, we adopt the definition of the objective QOL proposed in^6^: “Quality of life usually refers to the degree to which a person’s life is desirable versus undesirable, often with an emphasis on external components. […] In contrast to subjective well-being, which is based on subjective experience, quality of life is often expressed in more “objective” terms, describing the circumstances of a person’s life rather than his or her response to those circumstances”. It is related to the definition in^7^, where “quality of life is mainly considered as people’s being and doing and not only as being satisfied or having commodities”.

While there have been many studies of urban quality of life, they have tended to evaluate the city as a whole, neglecting the differences in well-being that occur within its borders. Such an approach is a valuable tool for policy makers, as it allows comparisons to be made between cities and their policies (e.g. some cities have a high quality of life due to the easily accessible health care, so more hospitals, doctor’s surgeries and pharmacies should be built in cities with lower QOL scores) but it ignores the fact that many aspects of quality of life have very local character, so without another measure, operating at a finer spatial scale, the problem remains (where exactly should they be located?). The literature discussing the urban quality of life at the micro level is still very limited, despite the clear need: tools for measuring local well-being are useful not only to policy makers, but also to entrepreneurs facing decisions on where to locate their investments and to people looking for a new place to live. There is no doubt that further research is needed.

The micro-scale indices of quality of life that have been developed so far represented various approaches. The studies^8,9^ took a microeconomic perspective, measuring the neighbourhood’s quality of life as a monetary value of its public facilities. However, although the proposed index took urban population density into account, it was only used as a variable in the model and not as a standardisation tool—the neighbourhood’s amenities were not calculated per capita. Studies^10,11^ have evaluated urban well-being as the weighted sum of the indicators at the district scale. The disadvantage of this approach is the large area covered by one analytical unit—the districts are so large that they are internally very diverse in terms of quality of life, making the results unreliable. Papers^12–14^ use buildings as units of the study. Nonetheless, there is often not enough data to cover the whole city with POI (Point of Interest) data or walking circles around buildings, so some of the amenities available in the city may not be included in the index.

Results from the literature typically show the highest levels of quality of life in the city centre and a gradual decline with distance from the centre^9,12–15^. Therefore, the primary hypothesis to be tested in the empirical part of this study is that Warsaw follows the same spatial pattern of high inner and low outer levels of QOL. Alternatively, some research reports clusters of high quality of life in various locations across the studied city, not necessarily in the central part^11,16^. Thus, the second hypothesis in this paper is that there are hot spots of exceptional living standards in Warsaw’s residential districts.

This paper introduces a new neighbourhood-level quality of life index (QOLI). The index is based on Moreno’s 15-min city concept^17^, which assesses the objective quality of life through proximity to urban amenities, goods and services, green spaces and transport infrastructure in analytical units of a 1 km^2^ grid cell. The main objective of the study is to provide policy makers with a reliable, easily accessible and interpretable tool to measure the well-being of urban residents and to visualise and evaluate its spatial distribution. Such an approach addresses both the problem of identifying resident’s needs and the question of where precisely to locate the desired solutions, which is a key to successful urban planning. The index is also used to measure the quality of life in Warsaw, Poland. The research question that goes beyond the study is whether Warsaw can be considered as a 15-min city that offers its inhabitants a possibly equal distribution of urban amenities.

The remainder of the paper is structured as follows. “Introduction” discusses subjective and objective perspectives on quality of life, existing urban quality of life indices and the 15-min city concept. “Related literature” introduces a new urban quality of life index, which is then empirically illustrated in “Local Quality of Life Index” using the city of Warsaw as an example. “Empirical example: Warsaw” discusses the results obtained and suggests policy implications, while "Conclusions" presents the conclusions.

## Related literature

### Subjective and objective quality of life (QOL)

The concept of quality of life is multidimensional and consists of both objective and subjective aspects^15,15,18–21^. The objective approach to measuring QOL focuses on the environment and objective living conditions, while the subjective approach represents people’s feelings^22,23^. Thus, objective indicators include employment status, income, housing conditions, access to urban amenities and many others; such variables usually collected by statistical offices or available through open-source data^23^. On the other hand, subjective measures are obtained through questionnaires in which residents are asked to rate their subjective happiness and life satisfaction, or to describe their perceptions and preferences regarding a given set of QOL components^4^. For example, the number of convenience stores in the neighbourhood would be an objective measure, while residents’ satisfaction with the access to convenience stores in their area would be a subjective indicator. Research has shown that although the subjective and objective quality of life are related, the correlation between them is weak, and therefore they should be treated rather as two separate concepts rather than different perspectives of the same phenomena^19^.

There is debate about whether subjective and objective measures of QOL should be mixed. Some research supports the view that successful policy-making should cover as many QOL dimensions as possible, preferably including both subjective and objective aspects^4,22,24^. However, there are two major barriers to this. First, objective indicators measure objective well-being, which is not the same thing as subjective well-being, as discussed above; they could be measured separately with different sets of indices and then combined afterwards for more effective urban planning. Secondly, the subjective approach has been widely criticised. The surveys used to obtain subjective indicators are expensive and time-consuming^12^, resulting in small numbers of respondents in scientific studies (e.g. the study sample in^25^ consisted of 200 Istanbul inhabitants in 2001^22^; surveyed 379 households in 2008^26^; used a QOL dataset of 6636 responses in 17 municipalities in 2017^27^; interviewed 500 inhabitants) and other difficulties in practical use for urban planning. They are unstable over time, with fluctuations not necessarily related to changes in objective living conditions; they depend on personality traits and life experiences, making assessments of two different people incomparable; finally, the assessments are rather emotional rather than cognitive, and thus unintelligible^20^. There is also the question of whether the scores obtained through surveys reflect true living conditions or reveal people’s unawareness of their environment^18^. Subjective indicators are used at an individual level, while at higher scales objective measures are more accurate^4^. In summary, objective indicators are more accessible, easier to standardise, and highly flexible for different levels of analysis; they are the best available measures^18^. Therefore, this paper focuses exclusively on the objective approach.

### Just city, needs-based city and 15-min city

The literature offers few ideas on how urban amenities should be distributed throughout the city. They are based on different assumptions: ‘*just city*’ is one in which facilities are distributed evenly across the space, the ‘*needs-based city*’ is the one in which they follow the needs of inhabitants, while the ‘*15-min city*’ is one in which they are accessible within a quarter of an hour. This is important for the spatial organisation of the city and for the quality of life.

The ‘just city’ approach promotes even distribution, so that every resident has equal access to public services, regardless of social or economic status, ability to pay or other requirements^28^. The concept emphasises the *equality of opportunities*^9^, and the reduction of inequalities in quality of life. The urban economics literature supports the concept of the ‘just city’. Brambilla et al.^9^ argue that an even distribution of urban facilities improves the overall quality of life, and therefore policies that promote more equitable access to amenities should be supported. Berliant et al.^29^ showed that, under certain conditions, an even distribution of public amenities forms an equilibrium. Achieving spatial justice in the city requires an equal distribution of resources, spatial benefits and opportunities^30^. The minor point, however, is that even equal distribution may not be sufficient to meet inhabitants’ needs—fair distribution is not always good enough allocation. This concept follows the philosophy of a universal city, where every place is attractive to everyone.

An alternative approach, the ‘needs-based city’, assumes that the distribution of urban amenities should depend on the particular needs expressed by residents living in different neighbourhoods, for example by locating more playgrounds and schools in the areas where families with children live^9^. However, this urban strategy can result in homogenous, specialised communities. As shown by^31^, this social segregation can create unproductive ghettos of low-skilled workers, leading to the complete collapse of the city’s productive sector.

The idea of the 15-min city was introduced by Carlos Moreno in 2016 and has been widely discussed in the literature^12,17,32–38^ as well as commonly implemented in planning policy in various forms (e.g. 20-min neighbourhood in Melbourne; 15-min policy in Paris; 45-min Singapore). The strongest evidence of the success of the 15-min city is the 2020 re-election campaign of the Mayor of Paris, Anne Hidalgo. Incorporating the 15-min plan helped her gain public support and win the election.

Ideally, in a 15-min city, urban dwellers should be able to meet all their daily needs and reach the essential amenities within a 15-min walk or bike ride from home; including both public facilities (such as schools, hospitals, or libraries) and private businesses (shops, restaurants, pharmacies etc.). Such an approach promotes social interaction between neighbours and their sense of belonging to the local community while supporting sustainable development and encouraging walking and cycling over driving. However, the 15-min city does not define the spatial resolution of the neighbourhood—cyclists will cover much more distance in 15 min than pedestrians; instead, to make the city more liveable, it encourages a rethinking of urban policies, promoting self-sufficient neighbourhoods and hyper-proximity to services. Interestingly, unlike the previous two concepts, it does not focus on the density of urban amenities, but on their accessibility.

The 15-min city concept was originally developed around four key principles: proximity, diversity, ubiquity, and density^39,40^. Proximity refers to short distances between the urban dwellers and the facilities, buildings, green spaces and any other places needed for convenient daily living. Diverse neighbourhoods are understood both as multicultural communities and as a mix of urban amenities that offer a wide range of goods and services to all citizens; ubiquity promotes equal access to public benefits regardless of individual characteristics, financial situation, or social background. Finally, density requires each neighbourhood to be inhabited by enough residents to make it profitable to open a business. In his new paper Moreno updates the list, replacing ubiquity with digitalisation^17^ to meet the modern needs in the emerging post-pandemic world.

The 15-min city embraces the ideology of ‘chrono-urbanism’, which states that the quality of urban life is negatively related to the amount of time spent travelling^17^. The 15-min city emphasises the need for cities to be people-centred^39^ by working at the micro level, making neighbourhoods the local centres of daily activity. It is about bringing activities to the neighbourhoods and rather than people to activities^33^.

Therefore, this study adopts a mix of two approaches. First, the ‘just city’ perspective promotes an even spatial distribution of urban facilities for equal and fair opportunities for each resident. It justifies an equal weighting of amenities in each location. Second, the 15-min city favours the accessibility approach over density-focused urban design.

### Urban quality of life

The last century has seen a significant increase in the proportion of people living in cities. Following the boom in global urbanisation, research on urban quality of life emerged in the hope of providing valuable tools for evaluating urban well-being and effective urban planning.

In general, two types of indices are used to measure urban quality of life—they differ in the spatial scale of the study. The macroscale approach aims to assess the city as a whole and usually uses the established methodology to rank cities according to their standard of living. Notable examples include the Urban Quality of Life Index, which is used to compare and evaluate Cuban first-tier cities^24^, the Arcadis Sustainable Cities Index, which ranks 100 global cities using 51 metrics^41^, the Urban Living Standards Index, which ranks 98 of the largest metropolitan areas according to their quality of life^42^, an index with preference-based weights, established upon differences in housing prices and wages, as well as the ranking of 253 urban counties^43^ or the ISO 37120:2018 ‘Sustainable Cities and Communities’ standard, which includes indicators for urban services and quality of life^44^. However, macroscale indices, as useful as they are, do not take into account intra-urban variations in quality of life. Some aspects of QOL have a very local nature and are therefore unevenly distributed across the city^20^.

On the other hand, the micro-scale approach focuses on a single city, and evaluates its districts or neighbourhoods. So far, researchers have conducted analyses at different spatial scales, such as street blocks^12^, group blocks^15^, exogenously partitioned *n* neighbourhoods^9^, city districts^11^, or even buildings treated as POI data or walking circles around them^13,14^. Fine spatial resolution allows comparisons to be made between distinct parts of the city, identifying areas where the quality of life is low and where policy makers need to take urgent action; however, the size, proportions and shapes of the analytical units also matter. Areas that are too large or irregular can themselves be very diverse, so that people living in the same unit can experience very different living conditions. For example, in the case of the Socioeconomic Well-Being Index, developed by Sánchez et al.^11^, the analytical units are the central districts in Madrid, while in some of them (e.g. the Retiro district) the internal spatial pattern is composed of both densely populated housing estates and large areas of green spaces. As a result, such a district could be considered as having a low standard of living due to high levels of air pollution, noise or traffic density, while the residents living near the park and outside of the housing estate do not suffer from such disadvantages. One of the alternative approaches is to consider the area within walking distance of households. The study areas of Wang et al.^14^ are circles with radii of 400, 800, 1200, and 1600 m centred on each housing estate. The approach presented is very promising as it ensures the accessibility of facilities located in the study areas. However, problems arise when there is not enough POI data to cover the entire city with circles—some urban features, located “in-between”, would not be taken into account, although they are undoubtedly of great use to the people living nearby. Therefore, in this study, the city is partitioned into 1 km^2^ square grid cells: small polygons of the same shape and size covering the entire study area. Each grid cell is considered to represent a neighbourhood, and facilities and urban features within 1 km of each other are within walking distance. It also approximates the neighbourhoods of a 15-min city—the average walking speed in the city is about 3–5 km per hour, so 1 km (or 1.4 km on a diagonal of the grid) is about a 15-min walk. The grid used in this study is the same as that used in the European Census data, which allows for reliable *per capita* analysis. The literature indicates that small grids of 1 km^2^, both square and hexagonal, are safe solutions to the MAUP (Modified Areal Unit Problem)^45^ and that this type of territorial division does not affect the estimation results^46,47^. Larger grids may interfere with the estimation, especially in cities of irregular shape.

Previous studies have used large collections of objective QOL indicators. Among the most commonly used measures are health indicators (e.g. number of physicians per 1000 residents; infant mortality rate, number of hospital beds per 1000 inhabitants), economic indicators (e.g. unemployment rate; income tax; % of income spent on food), crime indicators (number of serious crimes per 1000 residents; total crime rate; murder/manslaughter; criminal damage), climate and weather (e.g. average temperature, number of rainy days; total snowfall; humidity), demographic variables (e.g. % of non-white residents; population density and growth), quality of the environment (air or water pollution), access to urban facilities (e.g. proximity to urban amenities; recreational activity; tourism; restaurants per capita), education indicators (e.g. pupil/teacher ratio; % of children in secondary education; school drop-out rate) or housing standards (e.g. % dwellings with water/electricity; a number of rooms; a number of bathrooms; the age of housing)^48^. The full list of objective urban indicators to consider is too long to be exhaustive. In any case, it depends on the objective of the study, the spatial fragmentation and the availability of data. In this paper, quality of life is measured by the ability of the neighbourhood to meet all the daily needs of its residents. In this case, the availability of urban amenities is of primary importance.

### Existing indices of life quality

In the economic literature, the first notable micro-scale QOL index was developed by Roback^8^. He assumed that, given a set of neighbourhoods and the distribution of amenities within the city, economic agents will seek the location that maximises their utility. Thus, the revealed preference approach expresses urban amenity quality as the weighted sum of the monetary values of the city’s facilities. The weights are the prices of the amenities and are estimated through regressions on housing and wages. Extending this model, Brambilla et al.^9^ introduced an assumption of preferences for an equal distribution of urban amenities, resulting in an equity-adjusted QOL index. They applied their model to evaluate the quality of life in Milan, Italy on the neighbourhood scale. A different approach was proposed by Garau and Pavan^23^ who developed the Indicator of Smart Urban Quality (${I}_{SUQ}$), which evaluates six categories of indicators on the census area scale. The indicators are scored on a 5-point scale, from poor to excellent, against the standards set by the master plan. The performance of neighbourhoods is calculated as the average of the scores of their census sections. The sum of the scores forms sub-indices, one for each category; then, the ${I}_{SUQ}$ is then calculated as the sum of the six sub-indices. The index was then used to evaluate the quality of life in Cagliari, Italy. Another index, introduced by Şeker^10^ consists of 100 objective indicators grouped into three categories. The weight of each indicator is determined by a group of experts. The index is calculated for each district in Istanbul, as the weighted, standardised mean of the indicators. A similar approach was proposed by Sánchez et al.^11^ to measure the quality of life in the central districts of Madrid using environmental and socio-economic variables. The indicators were evaluated using Frechet’s Distance. The global index was calculated as the weighted sum of the indicators and then iteratively recalculated until convergence, taking into account the correlation between the indicators and the index. The Wang et al. index^14^ operates at a finer spatial scale, where buildings are the analytical units. The indicators, grouped into six categories, were used to assess the accessibility of public service facilities in the walking circles of the settlements. The weights given to the variables were based on the frequency of use of the facility. The overall index was the sum of the six sub-indices, one for each category. The methodology presented in this paper was implemented to evaluate accessibility to services within the Second Ring Road in Shijiazhuang, China. Also, index by Ye et al.^13^ focused on the POI data. They constructed a life convenience index to compare two Chinese cities, Guangzhou and Shenzhen. Eight categories of indicators were used and the variables were weighted according to the public surveys and the Analytic Hierarchy Process. The global indicator was calculated as the weighted sum of the eight sub-indices.

## Local quality of life index

### The general structure of QOLI

The Quality of Life Index (QOLI) proposed in this paper is based on eight categories of urban amenities, representing eight dimensions quality of life, collected for grid areas (Table 1). They express the neighbourhood’s ability to meet all the daily needs of its residents. These are: (i) shopping, (ii) dining, (iii) education, (iv) healthcare, (v) sports, (vi) transport, (vii) nature, and (viii) a large category ‘other’, which covers a long list of amenities not included elsewhere, such as services, leisure and entertainment facilities, crafting, financial facilities, cultural heritage etc. All of this data is POI available through web services such as Google Maps, OpenStreetMap, Baidu Map etc. for most places in the world. In this paper, the entire city is partitioned into grid cells, i.e. square-shaped, identical, adjacent neighbourhoods, each 1 km^2^ in size, roughly equivalent to a 15-min walk. This grid contains population data from the census. Locations of amenities (POI) are assigned to grid cells, resulting in a number of amenities in each cell. The index also refers to the neighbouring eight cells (queen design), which ensures that spatial autocorrelation and spatial dependence in urban systems, which are naturally continuous patterns, are dealt with.

**Table 1:** Categories and their facilities in each group (full list of additional facilities in Appendix 1).

Level category	Primary	Secondary	Tertiary	Additional
Dining	Fast-food	Restaurants	Ice cream Café	Bar Pub Biergarten …
Transport	Total daily number of bus and tram departures	Subway entrance	Parking Bicycle rental	Boat rental Car rental Taxi
Healthcare	Pharmacy	Doctor	Clinic Dentist	Hospital Veterinary Psychologist …
Education	School	Kindergarten	Library University	Driving School Language School Music School …
Sport	Soccer	Fitness	Basketball, Volleyball	Badminton Judo Tennis …
Shopping	Convenience store	Greengrocers	Bakery Supermarket	Shoes Clothes Health food …
Nature	Cell’s area covered by a park or a forest	“greenness” calculated with NDVI index	Meadow Water body (pond or river)	Hill Stone Flowerbed
Other	–	–	–	–

The QOLI index we propose is based on three assumptions: (a) per capita evaluation of the essential amenities, (b) less significant impact on the QOL of the establishments located in the second-order neighbourhood, and (c) the uneven importance of the facilities within each category. First, due to the uneven distribution of population in cities, the essential facilities should be calculated per capita, as other studies have shown^15,18,24^. However, this assumption holds as long as the amenity is visited regularly—these are “must-be” amenities; establishments used occasionally are usually too sparsely located to serve only the neighbourhood’s residents ("nice-to-have” amenities). For example, theatres, universities or hospitals will certainly attract people from all over the city; in such a situation, the per capita calculation would not be accurate, and the actual user population might be hard to estimate. In this case, the facility is treated as a pleasant addition to the neighbourhood’s diversity rather than as a prerequisite for a convenient daily life. Moreover, when a given type of facility is not present in residents’ closest neighbourhoods, they are willing to use facilities in the adjacent neighbourhoods, located still within walking distance from their homes^9^. Thus, the index takes into account not only the facilities located within the given cell, but also those located in the neighbouring cells, although they are given a lower weight.

Finally, the quality of life in a given neighbourhood depends mainly on a few types of features, while all the others are of lesser importance. For example, we expect people to value proximity to convenience shops more than to greengrocers or clothes shops, to trams or bus stops more than to underground stations, or to fast-food restaurants more than to pubs. The hierarchy of facilities may vary from place to place, depending on the local culture, the degree of coverage of the city with the preferred facilities, and the preferences and lifestyle of the city’s residents. Nevertheless, such an approach results in different implicit weights for different types of facilities within the same category. The highest weights are given to those facilities that fulfil four main conditions: (1) they are densely located throughout the city, (2) they are used for multiple purposes (e.g. soccer fields usually are simply grassy fields, thus any other kind of game could be played there; convenience stores sell bread and vegetables, thus could compensate for a potential lack of bakeries and greengrocers in the area), (3) they are used more frequently than the others, ideally on a daily basis (e.g. convenience stores) and (4) they must be accessible in a very short time when needed (e.g.. pharmacies).

The index, by design, is to reflect the hierarchy of inhabitants’ needs. At the individual level, these differ due to the diversity of tastes and preferences. The index can be derived for individuals, using the personal structure of needs, but when composed for aggregated preferences it should refer to generalised hierarchies of needs. This approach is related to the classic Maslow’s^49^ hierarchy of needs.

### Details of QOLI

The overall QOLI index is obtained by averaging eight sub-indices, one for each category—dimensions of life quality (rows in Table 1). The sub-index for the last category ‘other’ is intended to measure local business diversity and is described in detail further. The construction of the first seven sub-indices is as follows: first, the city is partitioned into neighbourhoods based on the grid coverage (here 1 km^2^). Secondly, the facilities are classified into one of four levels (columns in Table 1): primary facility (one type of POI, Eq. 1), secondary facility (one type of POI, Eq. 2), tertiary facilities (two types of POI, Eq. 3), additional facilities (many types of POI, Eq. 4). Each of these four levels defines one component of the sub-index i_cat_ (Eq. 5)—the generalised formulae are as follows:

1 $${i}_{cat, level\_1} = \lceil \frac{{n}_{cat,level\_1,cell}+{W}_{nb}\cdot {n}_{cat,level\_1,nb}}{quantile({n}_{cat,level\_1,cell}+{W}_{nb}\cdot {n}_{cat,level\_1,nb}, 0.75)}\cdot {W}_{1}\rceil$$

2 $${i}_{cat,level\_2}= {W}_{2}\cdot {n}_{cat,level\_2,cell}+{W}_{nb}\cdot {W}_{2}\cdot {n}_{cat,level\_2,nb}$$

3 $${i}_{cat,level\_3}= {W}_{3}\cdot {n}_{cat,level\_3,cell}+{W}_{nb}\cdot {W}_{3}\cdot {n}_{cat,leve{l}_{3},nb}$$

4 $${i}_{cat,level\_add}=min({W}_{add}\cdot {n}_{cat,level\_add}+{W}_{nb}\cdot {W}_{add}\cdot {n}_{cat,level\_add,nb} , {W}_{add\_max})$$

5 $${{i}_{cat}={i}_{cat,level\_1}+{i}_{cat,level\_2}+{i}_{cat,level\_3}+i}_{cat,level\_add}$$

where ${i}_{cat,level\_1}$ is the contribution to QOLI from the primary facilities, ${i}_{cat,level\_2}$ is the contribution to QOLI calculated using secondary facilities, ${i}_{cat,level\_3}$ comes from tertiary facilities, and ${i}_{cat,level\_add}$ represents the value of additional facilities. ${W}_{1}, {W}_{2}, {W}_{3}, {W}_{add},$ ${W}_{nb}$ are the weights given to the primary, secondary, tertiary, additional facilities, and neighbours respectively; ${n}_{cat,level\_1,cell}$ is the per-capita number of the primary facilities of a given category in the given cell and ${n}_{cat,level\_1,nb}$ is the sum of per capita primary facilities of a given category in all neighbouring cells; ${n}_{cat,level\_2,cell}$, ${n}_{cat,level\_3,cell}$ and ${n}_{cat,level\_add,cell}$ are dummies for each kind of secondary, tertiary, and additional facilities of the given category present in the analysed cell, while ${n}_{cat,level\_2,nb}$, ${n}_{cat,level\_3,nb}$ and ${n}_{cat,level\_add,nb}$ are the same for neighbouring cells (counted if those amenities are absent in the analysed cell); ${i}_{cat}$ is the value of the sub-index for one of the main categories: shopping, dining, education, healthcare, sports, transport, and nature (rows in Table 1). As expressed in Eq. (1–5), QOLI for a given grid cell is the weighted sum of POIs in the analysed cell and in the direct neighbourhood. Details of calculation of Eq. (1–5) are reported below.

The weights ${W}_{1}, {W}_{2}, {W}_{3}, {W}_{add},$ ${W}_{nb}$ and ${W}_{add\_max}$ are an important, but technical part of QOLI. Different weights express the uneven importance of types of facilities within a category and act as scaling factors of levels to make QOLI always in the range [0,10] independent of POI volume and number of inhabitants, and therefore comparable between different time periods and areas. The importance of urban amenities is defined in classification into levels (columns of Table 1). Changing the weights would decalibrate the index from the range [0,10]. In general, each sub-index ${i}_{cat}$ operates with integer and fractional parts. The integer part of the sub-index ${i}_{cat}$ corresponds exclusively to ${i}_{cat,level\_1}$, a value defined by the access to primary facilities. The fractional part of ${i}_{cat}$ describes the grid cell diversity and consists of ${i}_{cat,level\_2}$, ${i}_{cat,level\_3}$, ${i}_{cat,level\_add}$ components. The following weights (Eq. 6) are applied:

6 $${W}_{1}=9, {W}_{2}=0.3, {W}_{3}=0.2, {W}_{add}=0.1,{{W}_{add\_max}=0.3, W}_{nb}=0.5$$

**Figure 1:**
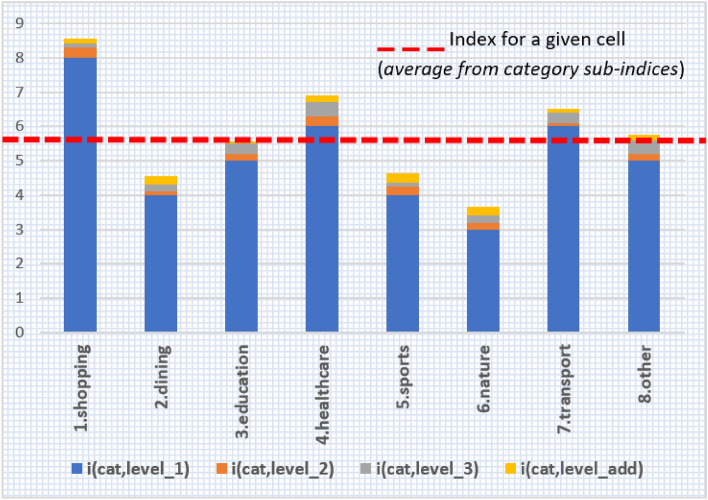
Construction of index for particular grid cell.

The justification of weights is as follows: ${W}_{1}=9$ scales the number of primary level facilities ${i}_{cat,level\_1}$ to an integer from the set {0, 1, …, 9}. The weights ${W}_{2}=0.3, {W}_{3}=0.2, {W}_{add}=0.1$ make the ${i}_{cat,level\_2}$, ${i}_{cat,level\_3}$, ${i}_{cat,level\_add}$ positive numbers no larger than 0.3, 0.4, 0.3, respectively – they make a fractional part of the sub-index ${i}_{cat}$. The fractional part is usually no greater than $max({{i}_{cat,level\_2})+max({i}_{cat,level\_3})+max(i}_{cat, level\_add})=0.3+0.4+0.3=1$. ${W}_{nb}$ is the weight of neighbours, which acts as a distance penalty—with W_nb_ = 0.5, only half (50%) of the amenities of neighbouring cells are taken into account, mainly due to reduced convenience and accessibility at a greater distance from a given POI. Therefore, the i_cat_ index in the range of 0–10 is a composite of the main part (integer part)—value between 0 and 9, coming from the primary level, and the minor part (fractional part)—value between 0–1, coming from the second, third and additional levels. The QOLI is an average of the category sub-indices (Fig. 1).

Not only the weights W are responsible for scaling of the volume of POIs per capita. The component i_cat,level_1_ contains the benchmarking with the 3rd quartile. The expression $quantile({n}_{cat,level\_1,cell}+{W}_{nb}\cdot {n}_{cat,level\_1,nb}, 0.75)$ (in Eq. 1) is the 75th percentile of the values ${n}_{cat,level\_1,cell}+{W}_{nb}\cdot {n}_{cat,level\_1,nb}$- this is ¾ of all units in analysed and neighbouring cells, weighted 100% in the analysed cell and 50% in other cells due to distance. The construction of individual subindices is explained below, while the discussion of index design was presented in Appendix 5.

**Figure 2:**
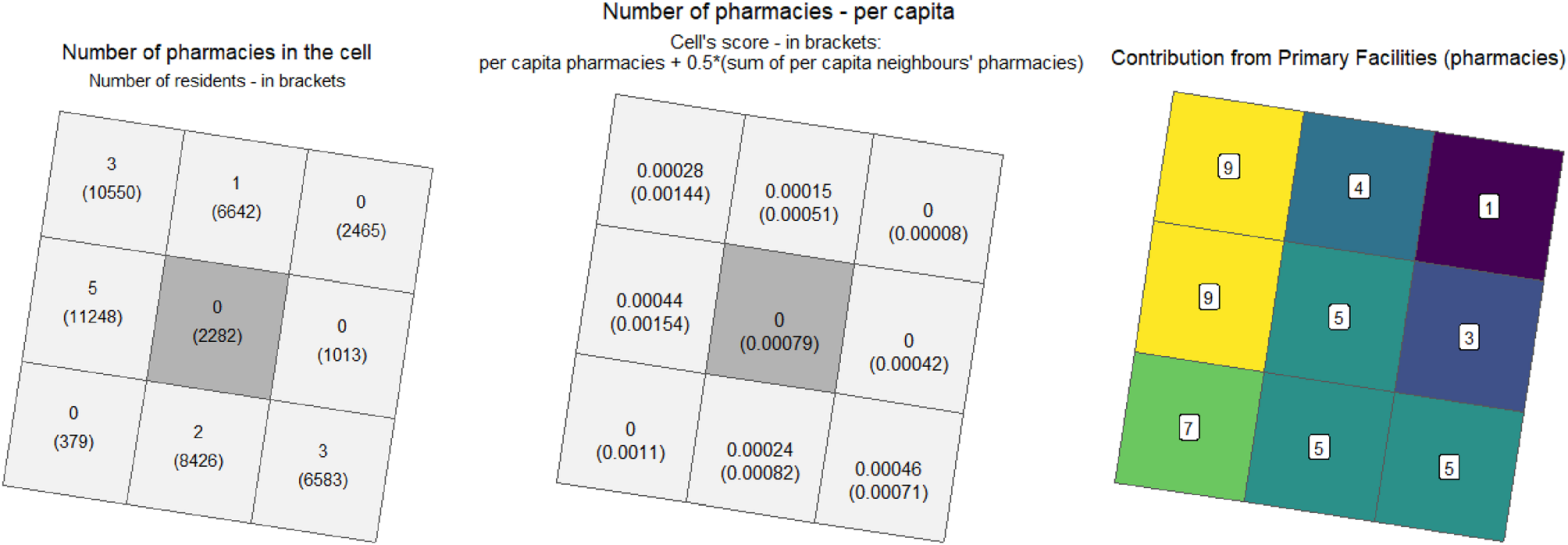
An example of the calculation of ${i}_{health, 1}$. In this category, pharmacies are the primary facilities. The plots present nine grid cells. (**a**) A number of pharmacies in each cell. A number in brackets represents the cell’s population. (**b**) *Per capita* number of pharmacies in each cell. The cell’s *score*—written in brackets—is calculated as the sum of the per capita number of pharmacies in the cell and half of the sum of the neighbouring cells’ *per capita* pharmacies. (**c**) The 75th percentile of scores obtained by all cells in the city was 0.001518784. Thus, all cells with the higher score were assigned the value 9. The rest of the cells were assigned the values proportionally, according to their scores. The values were rounded up to the next greater integer.

*Primary facility component:* It is based on the *per capita* number of primary facilities in each cell. According to Eq. (1), it sums the number of POIs per capita in a given cell and ½ of the POIs per capita in 8 neighbouring cells. Secondly, it calculates the Q3 (75% percentile) of this score in all cells and considers it as a benchmark—all the cells with a score higher than this value are assigned a value of 9. For all the cells with scores below this threshold, ${i}_{cat,1}$ is calculated using Eq. (1), essentially dividing the score by the threshold and multiplying by the weight = 9. Finally, the outcome is rounded up ($\lceil value \rceil$) to the next higher integer. As a result, ${i}_{cat}$ is a positive integer with values in the set $\{0, 1, 2, ...,9\}$. Note that the zero value is obtained only if there are no primary facilities neither in the cell nor in the surrounding neighbourhoods; the maximum value is assigned when the neighbourhood has relatively many primary facilities when compared to other neighbourhoods. Figure 2 illustrates how the ${i}_{health, 1}$ component is calculated. All components conditionally refer to the first-order (contiguity, common-border) neighbourhood—if amenities are missing in the analysed cell, they are searched for in the surrounding cells. Overall, amenities are primarily counted in 1 km^2^ grid, and conditionally in 8 km^2^ in neighbourhoods.

*Secondary facility component*: The group of secondary facilities comprises only one type of facility (Table 1). Thus, for each cell, one checks whether this facility is present there or not (i.e. one neglects the total number of establishments, being interested exclusively in a binary variable indicating the presence or absence of a given facility). The value associated with secondary facilities is described by Eq. (7):

7 $${i}_{ cat,2}=\left\{\begin{array}{c}0.3 \,if \,at \,least \,one \,object \,is \,located \,in \,the \,given \,cell \\ 0.15 \,if \,objects \,are \,only \,in \,the \,neighbouring \,cells \\ 0 \,otherwise\end{array}\right.$$

The calues in Eq. (7) are an outcome of multiplying the dummy variable by the weight. If the number of POIs of a given category in a given cell is > 0, then n_cat, level_2, cell_ = 1 and when multiplied by W_2_ = 0.3 yields i_cat,level_2_ = 0.3. The neighbouring cells are then ignored. However, if the analysed cell has no POI of this category, the neighbourhood data are taken into account. If a number of POIs of a given category in any neighbouring cell is > 0, then n_cat, level_2, nb_ = 1 and when multiplied by W_2_ = 0.3 and W_nb_ = 0.5 gives i_cat,level_2_ = 0.15. If neither the cell nor the neighbours have given POI, the index = 0.

The results are scaled: the cells could actually be assigned one of the values $\{0, 0.15, 0.3\}$: cells, whose residents have no access to the facility (no facility in their cell and in the neighbouring cells) are assigned the value 0, cells obtain the value 0.15 when the access is impeded (i.e. the facility is further away from the households, only in neighbouring cell), and wherever the facility is available closely (in their cell) the value becomes 0.3. The difference in values for cells with secondary facilities located *close* (i.e. within the neighbourhood) and *far* (i.e. in the neighbouring cell) acts as a distance penalty; the residents are less satisfied with their living conditions if they have to walk long distances to reach basic amenities.

*Tertiary facilities:* the construction of this component is very similar to the one for the secondary facility. However, this group includes two kinds of facilities (Table 1), resulting in the following three possibilities: (i) each of them could be present in the cell, (ii) missing in given cell but present in one of the neighbouring cells, or (iii) missing both in the analysed cell and neighbouring cells. Thus, the scale also differs. In the Eq. (3) the n_cat, level_3, cell_ takes the value of 2 if both amenities are located in the given cell, 1 if one of the two amenities is located in the given cell and 0 otherwise; similarly, n_cat,level_3,nb_ corresponds to 2 if both amenities are not present in the given cell but both can be found in one of the neighbouring cells, 1 if one of the two amenities is missing in the given cell but it is present in the neighbouring cells, and 0 if both amenities are missing both in the cell and the adjacent cells. Thus, ${i}_{cat,3}$, after weighting n_cat,level_3_ with W_3_ = 0.2 takes values from the set $\{0, 0.1, 0.2, 0.3, 0.4\}$.

*Additional facilities:* This level (group) represents all the facilities that belong to the given category, but are not classified as amenities of primary, secondary or tertiary importance. This is because they are not frequently used, not located densely enough, and not stated as important by the city’s citizens, or for some other reason that the researchers or policymakers found accurate. They account for the quality of life by increasing the neighbourhood’s diversity and providing more options for the residents. The value of ${i}_{cat,add}$ (Eq. 5) is upper-bounded by ${W}_{add\_max}$ to ensure that very diversified cells (with many additional facilities) still obtain rather low scores in the category if primary, secondary, or tertiary facilities are missing. In Eq. (4), n_cat, level_add,cell_ is the number of kinds of facilities that are located in the given cell and n_cat, level_add,nb_ is the number of those facilities that are not situated in the given cell but are present in at least one of the neighbouring cells. The weight W_add_ = 0.1 divides the number of facilities types by 10 in a given cell and by 20 (W_add_* + W_nb_ = 0.1*0.5) in neighbouring cells. Value of i_cat, add_ is 0.3 or less—Eq. (4) defines i_cat,add_ as a minimum from weighted amenities or W_add_max_ = 0.3. If weighted amenities are a large component, i_cat,add_ is limited to W_add_ max_, if there are no amenities, i_cat, add_ is close to zero (and below 0.3). The sub-index ${i}_{cat,add}$ takes values from the set $\{0, 0.05, 0.1, 0.15, 0.2, 0.25, 0.3\}$. The weight ${W}_{add\_max}=0.3$ is only used to restrict the values of sub-indices to the interval [0,10]. For example, there are four additional facilities in the category ‘dining’: bar, pub, beer garden and food court. The cell with two bars, one pub and no beer garden or food court would be given the value n_cat, level_add,cell_ = 2(because it contains two out of four kinds of facilities). If in the neighbouring cells one could find a bar, a food court and no pubs or beer gardens, so that n_cat, level_add,cell_ = 1 (because there is a food court absent in the previous cell, and the bar overlaps with bars from the previous cells, thus this cell contains only one new facility compared with the previous cell). This cell’s final score would be ${i}_{dining,add}=min(0.1\cdot 2+0.05\cdot 1 , 0.3)=min(0.25, 0.3)=0.25$. On the other hand, the cell containing all four additional dining facilities would be given the value ${i}_{dining,add}=min(0.1\cdot 4+0.05\cdot 0, 0.3)=min(0.4, 0.3)=0.3$.

*Category ‘other’:* The purpose of the ‘other’ category is to capture all the objective indicators related to accessible amenities that do not belong to any of the previous categories. It includes the facilities that are not used daily and their relative importance depends on residents’ preferences (which are heterogeneous on the city-wide scale, varying from person to person). Therefore establishing different weights would be unreasonable and would bias the index. Thus, QOLI treats this category as a measurement of the neighbourhood’s diversity: for each cell, the number of types of facilities was calculated (i.e. regardless of how many establishments of this kind there are; what matters is whether a given facility type—a bank, a theatre, a historical site, etc.—is present in the neighbourhood or not). Then, the relative diversity was calculated by dividing the obtained number by the highest number acquired in the city; the results are multiplied by 10, so that their range is [0,10], the same as of other sub-indices. For the full list of amenities and neighbourhood features included in this category, see Appendix 2.

*Index for the whole category:* Index i_cat_ is the total of subindices for each level (i_cat,level_1,_ i_cat,level_2_, i_cat,level___3,_ i_cat,level_add_). Cells obtain zero value only if there are no facilities of the given category neither within the cell nor in the neighbouring cells; the value of 10 is given solely to the neighbourhoods, whose residents have relatively high access to primary facilities (at least as high as 75% of city’s neighbourhoods) and many options regarding other establishments in the category (i.e. the neighbourhood is very diversified, and values of *i*_*cat,2*_*, i*_*cat,3*_ and *i*_*cat*, add_ are max).

*Main QOLI index:* The main Quality of Life Index QOLI is calculated as the arithmetic average of eight indices for each category:

$$i=\frac{{i}_{shopping}+{i}_{dining}+{i}_{education}+{i}_{healthcare}+{i}_{sports}+{i}_{nature}+{i}_{transport}+{i}_{other} }{8}$$

Obviously, through averaging the main index loses its simple interpretation. Instead, it provides a general overview of the distribution of quality of life in the city, enabling policymakers to identify areas with the lowest living standards understood as having no direct access to urban facilities. Category sub-indices indicate the cause of problems, suggesting an appropriate course of action. QOLI refers to ‘just city’ by assuming equal weights for amenities in different locations and to ’15 min city’ by looking at accessibility, and not only the density of POI. All the dimensions of the 15-min city’ concept are covered by the index: it represents a proximity-based approach, promotes densely located, multi-functional facilities, and accounts for accessible and efficient public transportation and a green environment.

## Empirical example: Warsaw

### Study region

**Figure 3:**
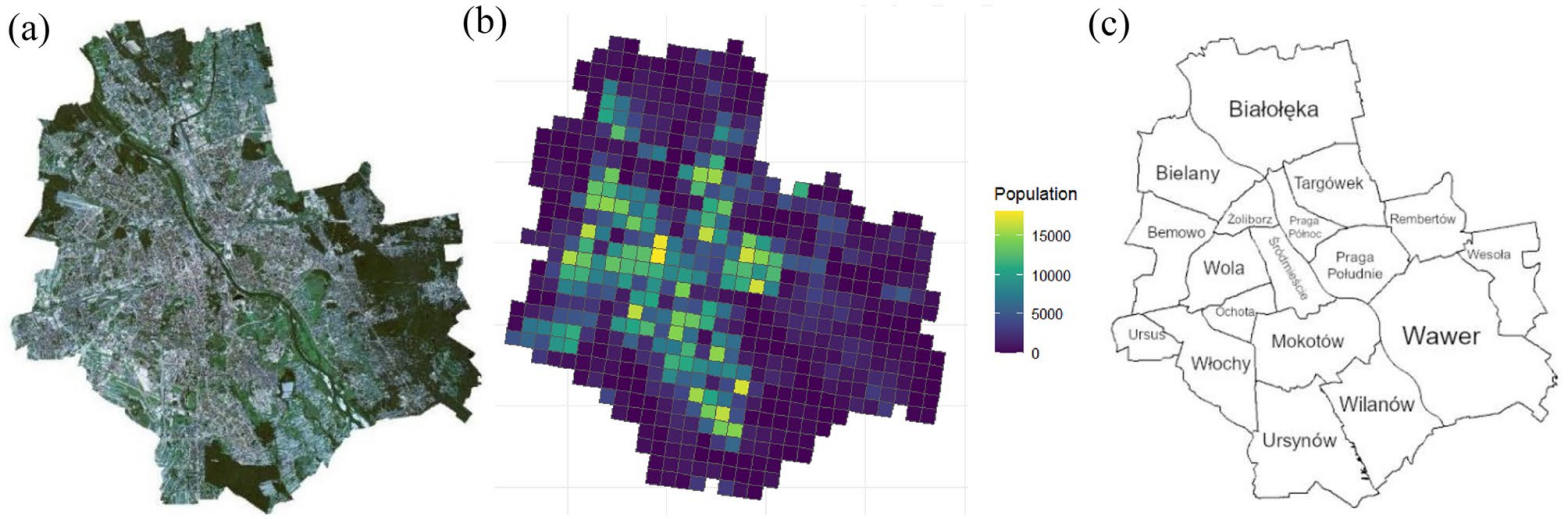
Warsaw: (**a**) satellite imagery, (**b**) population density, (**c**) districts.

The study area is Warsaw, the capital and largest city of Poland. The city is located in Mazowieckie voivodeship, in the country’s east-central region, in the centre of Mazovian Plain (52° 13′ 56″ N, 21° 00′ 30″ E). The average height is 100 m above sea level; the Vistula River runs through the heart of the city. Warsaw’s residents have access to many green spaces, including local parks, historical parks located in the city centre and vast urban forests, covering southern, eastern and northern parts of Warsaw, mostly along the city’s borders (Fig. 3a). Warsaw is home to 1.86 million people (Fig. 3b), living in 18 districts (Fig. 3c). The total area is 517.2 km^2^. The city is part of the larger metropolitan area, with a residential population estimated at 3.1 million, being the 6th most populous city in the European Union. Warsaw is an important academic, political, economic and cultural centre. This is an interesting case study of a post-socialist city, fully rebuilt after 2nd World War. The urban strategy is to achieve “convenient locality” by developing a compact city, with a polycentric and hierarchical functional and spatial structure, with a network of district and sub-district centers concentrating the offer of services and possibly low differences between districts^50^. Natural conditions, flat terrain, protected forests around, and lack of hills, mountains and lakes made it relatively easier to develop the desired urban shape.

### Data

Four sources of data have been used in the study. First, the grid data concerning population was retrieved from the Polish 2021 census (Fig. 3b), collected and shared publicly by GUS (Statistics Poland). The same grid population data are available for the whole European Union from the Eurostat. The dataset included the number of residents living in each of 601 grid cells, along with its decomposition into age groups (number of people aged 0–14 years, 14–65 years, 65 + years) and gender (number of female and male residents). Second, the Normalized Difference Vegetation Index (NDVI) was calculated based on satellite imagery published by the European Space Agency (Sentinel-2 mission) (Fig. 3a). The data consisted of three imagery tiles, collected on 1 July, 27 August 2022 and 6 September 2022. Third, the information about public transport timetables, and bus and tram stop locations were included in the General Transit Feed Specification (GTFS) files provided by OpenMobilityData on 29 September 2021. This was used to calculate the annual total number of departures from each stop. Fourth, all kinds of urban amenities and POIs, including shopping facilities, restaurants, educational objects, healthcare facilities, sports facilities or public service facilities were derived from OpenStreetMap (data collected on 26 March 2023). More details on data processing, R codes and datasets are in Appendix 4.

## Results

**Figure 4:**
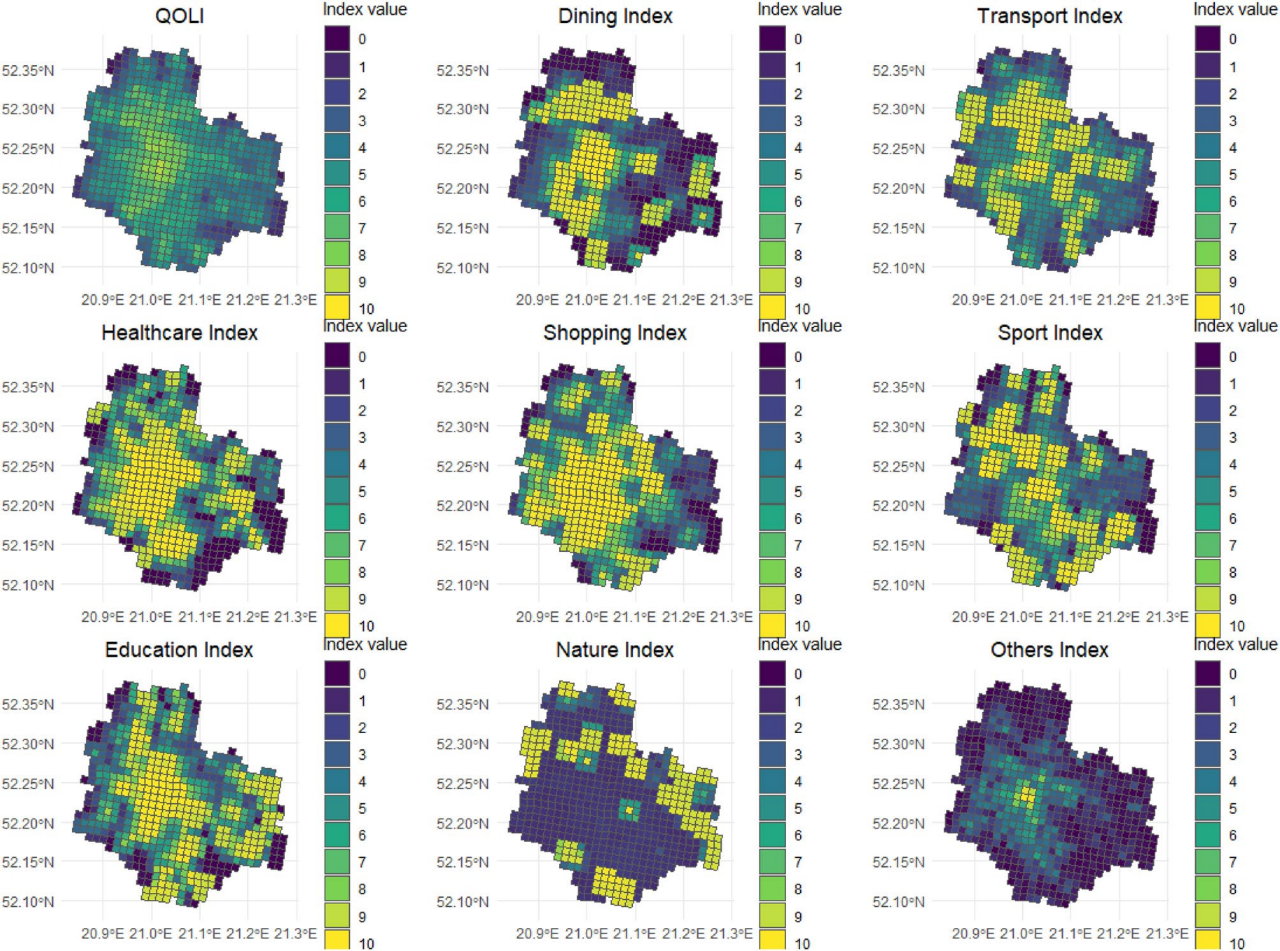
Distribution of quality of life in Warsaw. The presented indices were based on per capita values. A set of indices based on nominal data is available in Appendix 3.

The analysis described in “Related literature” has been run for Warsaw city. The distributions of values for all sub-indicators are presented in Fig. 4.

The study revealed that the quality of life in the central part of the city is significantly higher than in the outer districts, presenting a typical high-inside, low-outside pattern. The lowest QOLI values were reported along the city’s borders, as well as on the east and northernmost sides of Warsaw. However, such results could be partially explained by the presence of vast forests located in these areas.

The spatial patterns of sub-indices indicate that Warsaw is composed of a collection of specialized neighbourhoods. While the city centre performs high in all categories except for the natural environment, all the other districts (Table 2) excel at some aspects of QOL and fail to provide sufficient facilities for others. Hence, the residents of the central district (Śródmieście), and selected first-ring (Ochota and Praga Północ) and second-ring (Włochy) districts enjoy the rich dining services; the best access to transport infrastructure is provided in the central district (Śródmieście), and a few first-ring (Praga Północ, Praga Południe) and second-ring (Włochy, Targówek) districts. The distribution of medical facilities is surprisingly even throughout the city; however, peripheral neighbourhoods suffer insufficient access to healthcare. Shops are located mostly in the central and selected first-ring districts (Śródmieście, Ochota, Mokotów, Wola), missing on the east side. There are clusters of sports facilities located in various parts of the city, notably in the southern, outer neighbourhoods. Schools are easily accessible in the central and selected first-ring districts (Śródmieście, Żoliborz, Praga Północ and Mokotów). The values of the nature sub-index peak in the peripheries, coinciding with the locations of the city’s largest green spaces. The results within the category ‘others’ indicate that central district Śródmieście is the most diversified part of Warsaw (with respect to accessibility to facilities); the diversity gradually decays with distance from the city centre. The summary of districts’ performance in each category is presented in Table 2. The average value of QOLI by districts is 5.35. QOLI value of 6 divides, in general, districts into two groups: central and first-ring districts with QOLI > 6 and second- and third-ring districts with QOLI < 6.

**Table 2:** Average values of QOLI and sub-indices in Warsaw’s districts (sorted decreasingly by QOLI within consecutive rings).

Ring	District	QOLI	Dining	Transport	Health	Shopping	Sport	Education	Nature	Others
Central district	Śródmieście	**8.25**	**9.96**	**9.22**	**9.87**	9.85	8.45	**9.96**	1.53	**7.16**
First ring	Praga-Północ	7.17	7.02	8.62	9.59	8.99	7.62	8.38	3.72	3.41
	Żoliborz	6.92	6.39	7.09	9.29	8.07	**8.75**	8.76	3.61	3.40
	Ochota	6.85	9.2	7.45	9.9	**9.97**	5.22	6.83	1.48	4.73
	Wola	6.39	7.54	6.16	9.19	9.09	5.39	7.89	1.42	4.47
	Praga-Południe	6.19	6.32	7.69	7.18	8.34	6.64	7.22	2.98	3.20
	Mokotów	6.19	5.43	6.69	7.48	9.42	7.19	8.06	1.92	3.31
Second ring	Targówek	5.91	6.14	8.37	7.27	8.09	4.58	4.39	**6.48**	1.96
	Włochy	5.45	6.94	7.53	6.96	8.42	4.56	3.78	3.76	1.66
	Bielany	5.15	5.45	7.21	5.28	4.57	6.86	4.21	6.28	1.31
	Ursynów	4.89	4.69	3.62	5.56	5.81	6.10	6.48	5.27	1.55
	Wilanów	4.23	3.42	5.65	*3.11*	5.20	7.68	5.34	2.38	1.07
	Białołęka	4.02	3.28	4.53	4.77	5.06	4.79	5.09	3.74	0.86
	Wawer	3.97	2.63	4.19	5.29	4.00	4.45	6.04	4.26	0.86
	Bemowo	3.97	2.05	5.57	4.73	6.59	4.18	3.75	3.02	1.84
	Rembertów	3.80	*1.40*	4.38	5.28	4.54	4.91	4.59	4.37	0.90
Third ring	Wesoła	3.80	3.65	4.08	3.48	*1.71*	*2.14*	7.24	7.35	*0.71*
	Ursus	*3.07*	2.50	*2.92*	3.61	6.62	2.73	*2.92*	*1.38*	1.88

In bold are the maximum values, in italics are the minimum values.

The situation in some districts raises concerns: in Ursus and Wawer residents not only are not provided sufficient amount of basic facilities (restaurants, schools, and sports centres in Ursus; restaurants and shops in Wawer) but also cannot easily reach establishments located elsewhere, as their neighbourhoods lack access to public transport. Urban planners should equip these two districts with more bus and tram stops and more frequent connections, as well as implement policies favouring new business openings (particularly restaurants and shops). On the other hand, many districts are not superior in any category; perhaps including them in new policies would contribute to a sense of equality for all city residents. Nonetheless, at this moment Warsaw cannot be called a 15-min city, as the basic assumption of this planning model is violated: essential facilities and green spaces are not accessible in all neighbourhoods. In a 15-min city, the values of QOLI as well as of its sub-indices would be high (greater than 6) or medium (greater than 3) in all the neighbourhoods. As demonstrated in Fig. 3 and Table 2, Warsaw does not fulfil these criteria.

**Figure 5:**
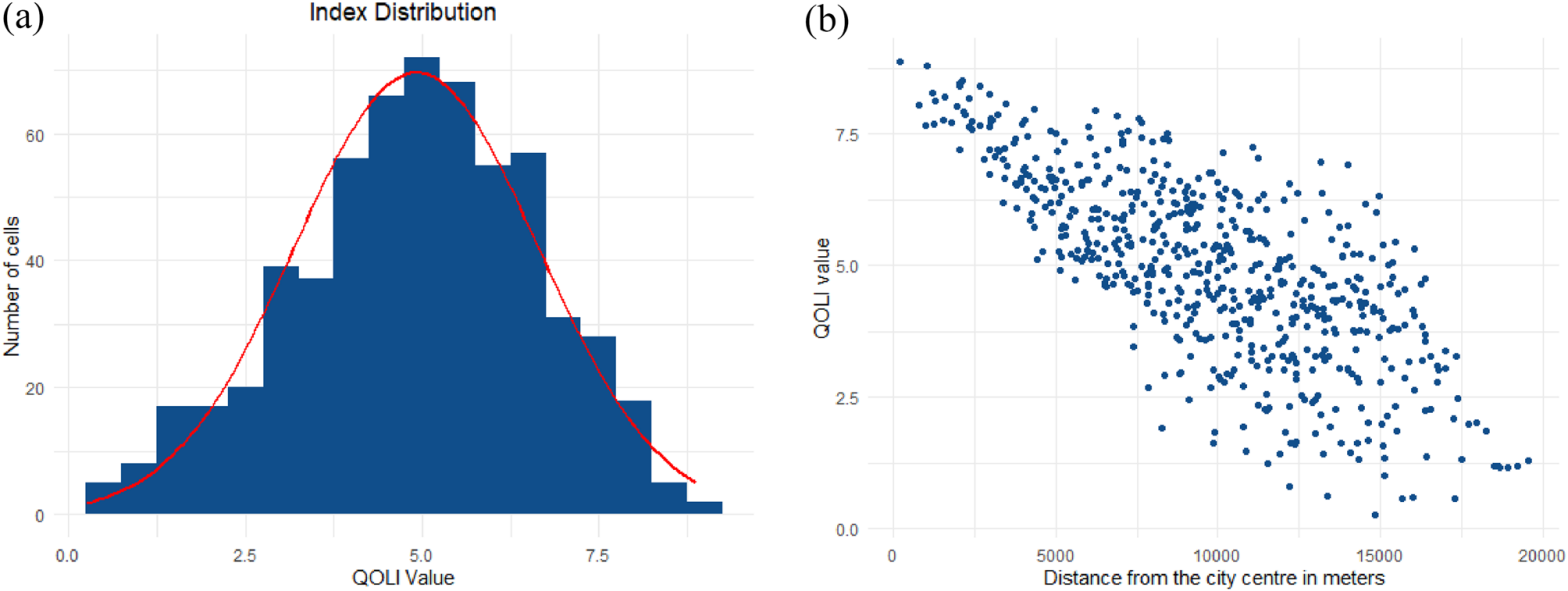
(**a**) Histogram of QOLI values, (**b**) Relationship between QOLI index and the distance from the city centre in meters.

The mean value of QOLI by individual cells reached 4.91, with a standard deviation of 1.72 (Fig. 5a). The study revealed a strong negative correlation between the local quality of life in Warsaw and the distance from the city centre, with the correlation coefficient $r=-0.7$ 3 (Fig. 5b).

## Discussion

The results obtained in the previous section are not surprising. The highest quality of life in the city centre and gradient-like descent with distance appeared in previous studies^9,12–14^, although in some of them, such pattern partially originates in the neglect of differences in population density throughout the city^13^. Nonetheless, the main hypothesis of this paper that the central districts are characterized by the highest levels of QOLI, while its lowest values can be found in the peripheral neighbourhoods cannot be rejected. On the other hand, the second hypothesis concerning the existence of hot spots of high QOLI that are dispersed around the city is rejected as there are no high-life-quality clusters outside the central district.

The distributions of the QOL index and the corresponding sub-indices are insightful not only to the policymakers but also to entrepreneurs and to the people looking for a new place to live in. Urban planners can examine, which neighbourhoods need new policies or public services; spatial patterns of QOL components suggest possible new business locations, profitable due to relatively low competition compared with benchmarks from more successful places. It is expected that typically migrations will be directed towards the neighbourhoods with the possibly high quality of life at given prices of housing^51^.

Clearly, one of the limitations of this study is the subjective choice of considered variables and their corresponding weights. However, the preferences concerning QOL aspects tend to be spatially heterogenic, depending on the local culture and the residents’ habits, making it impossible to establish a universal, fit-for-all set of weights; instead, the purpose of this study was to propose a general approach to measure the quality of life, conducting the analysis as rigorously as possible, while arranging the indicators in the reasonable order, supported both by the literature and the common sense. Nonetheless, the suggested methodology is highly replicable: it does not require advanced statistical tools, the data it uses are easily accessible and it is not computationally intensive. A predefined hierarchy of facilities, established externally, enables comparison of the distribution of quality of life in cities; it would not be possible in the case of different sets of weights in each city. However, for purposes of policy making, we recommend local authorities determine their city’s residents’ preferences regarding the relative importance of the quality of life components through a set of surveys, conducted on a city-wide scale. The results could help establish more accurate weights for the case of the specific city, making the outcome more reliable. Another approach could include combining the QOL index proposed in this paper with the set of subjective indicators (such as satisfaction with family life, marriage or free time) for a deeper understanding of the concerns that residents face daily, influencing their well-being and quality of life.

**Figure 6:**
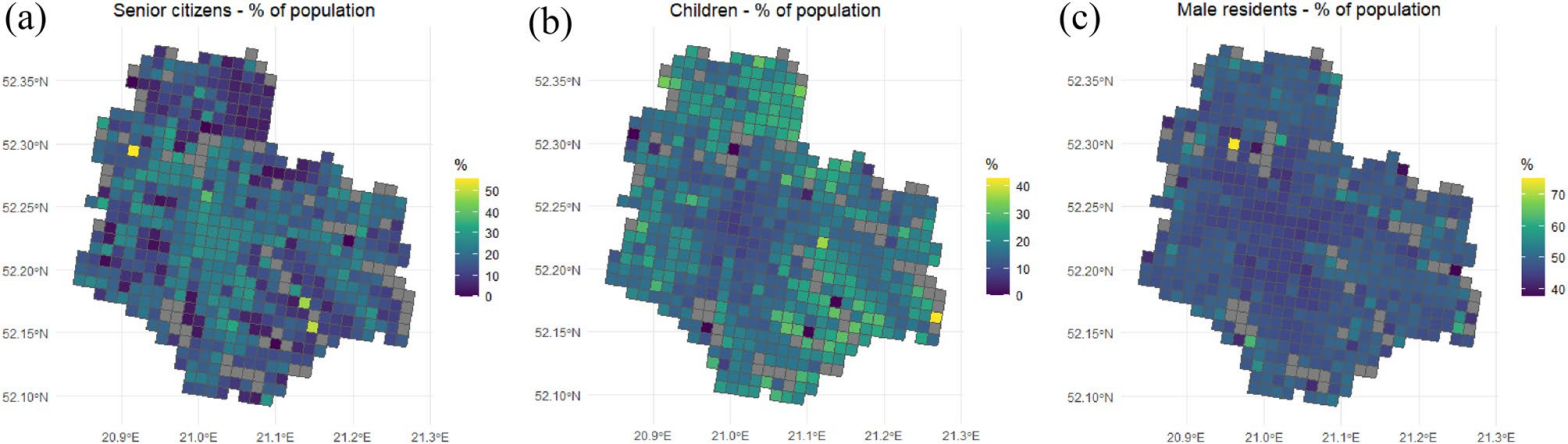
(**a**) Senior citizens as the percentage of the total cell’s population, (**b**) Children as the percentage of the total cell’s population, (**c**) Male residents as the percentage of the total cell’s population.

The ‘*just city’* approach applied in this study encourages even distribution of the facilities and services throughout the city; hence, in contrast to the ‘*need-based’* urban planning, it is possible that residents living in some neighbourhoods are not provided with enough essential facilities due to their particular needs (e.g. senior citizens require easy access to healthcare, children need schools, etc.). Naturally, a question arises as to what extent this decision affects the results and credibility of the study. To examine this issue, the spatial distribution of senior citizens, children and male residents was analysed (Fig. 6). Apart from Białołęka and Wilanów, two peripheral districts characterised by a notably younger population structure, the analysis revealed no significant differences in the relative distribution of residents representing these groups (the outlying cells have very low population density). Unfortunately, the other demographic data—e.g. regarding ethnic groups, residents with disabilities or religious affiliations—is not available at this moment.

Lastly, quality of life is a subjective phenomenon, heavily dependent on the residents’ perceptions and preferences. In addition to the regularly conducted questionaries, it would be worthwhile to actively include the citizens in the QOL-oriented urban policy-making. Typically, the residents are well-informed about the ongoing situation in their neighbourhoods and can alert the authorities in case of alarming circumstances they live in, so that immediate action can be taken. Alternatively, one may consider cross-validating the results with wealth or income data. However, they are still poorly available on low aggregation level, therefore approximations from nighttime-light data, satellite imaginaries or spectral data can be used. Recent literature develops linkages between those data and inequalities^52^, poverty^53^ and well-being^54^. Such an approach would help to enhance the local quality of life and make the city more liveable.

## Conclusions

In this paper, a new objective index for measuring the urban quality of life on the neighbourhood scale was presented. The Quality Of Life Index (QOLI) is based upon the urban planning concept known as the ’15-min city’, evaluating living standards by proximity to the amenities, services and green spaces. It consists of eight categories, representing eight dimensions of urban quality of life: dining, transport, healthcare, education, sport, shopping, nature and the category ‘others’ including crafting, financial facilities, cultural heritage, services, leisure facilities and more. Its construction requires partitioning the city into (1 km^2^) grid cells, determining the values of eight sub-indices (one for each category) in every cell, and averaging them using arithmetical mean. The sub-indices are calculated by finding the total number of amenities located in the cell and 8 surrounding cells and computing the weighted sum of their contributions to the quality of life; the establishments visited daily and located densely throughout the city are given the top priority and calculated per capita. The weights are established through the predefined hierarchy of facilities, which reflects the general social needs according to Maslow.

The empirical analysis of the quality of life in Warsaw, Poland revealed the typical pattern of high-inside low-outside quality of life. The highest living standards were noted in the central and first-ring districts, while the lowest were in the exterior districts, located along the city’s border. Moreover, it appears that Warsaw is composed of a collection of specialized neighbourhoods: typically, the grid cells’ performance was excellent in some of the QOLI categories and significantly worse in others. The attention of urban planners should be directed to the peripheral neighbourhoods, which lack both essential facilities and access to public transportation; new policies are needed to enhance the well-being of their residents. Lastly, the quality of life in Warsaw is strongly negatively correlated with the distance from the city centre, with the correlation coefficient of $r=-0.7$3.

The geodesign of the ‘15-min city’ promotes hyper-proximity to essential facilities, green spaces and public transport, so that urban dwellers can meet all their daily needs within a short walk or bike ride from home. The ‘15-min cities’ are made up of independent, diversified neighbourhoods, whose residents form micro-communities with a sense of belonging and daily interaction. The QOLI in a 15-min city is highly similar in all locations, as the accessibility to the urban amenities included in the QOLI should be equal, independently of centrality. As most of Warsaw’s neighbourhoods are rather specialized than diversified regarding access to facilities and services, Warsaw today is not a 15-min city.

The methodology presented in this paper provides valuable tools for urban planning. The index described in “Local quality of life index” exposes not only the spatial distribution of quality of life throughout the city but also the pattern of its components so that the cause of low living standards for some neighbourhoods can be quickly identified and the appropriate action taken. The construction of the index is simple and easily adaptable to the local circumstances—i.e. the preferences of the city’s residents or the goals set by local authorities—making it practical and easy to implement. Those preferences are expressed in the order of amenities to be included in the index (as in Table 1). Moreover, studies using grid cells as analytical units are still very uncommon, whilst measures built upon relatively small areas, equal in shape and size, are very precise and provide comparable results.

Quality of life is a complex concept and cannot be measured with a single tool completely. While in recent years, the problem of QOL has gained much attention, with more data being gathered and new measures emerging, more research is still needed to explore QOL dimensions, their relative importance and spatio-temporal stability. Hopefully, the combined efforts of researchers and urban planners eventually will manage to not only understand the concept of QOL better but above all improve overall urban well-being, bringing us closer to the common goal: liveable cities, where everyone is equally satisfied with their living conditions, regardless of their social, ethnical or economic status. Providing complete, enjoyable neighbourhoods is surely the first step. The index does not refer to negative externalities generated by air pollution, traffic, noise, crowds, prices of housing etc.^55^. They are usually considered the components of subjective well-being^56,57^ and their spatial distribution is ambiguous^58,59^, and availability at a fine scale is limited.

### Supplementary Information

Supplementary Information.

## Data Availability

QOLI index is fully replicable for a majority of world locations. R codes for calculations of the index are available at the GitHub repository at https://github.com/e-dobrowolska/QOL-paper. The R codes include data scrapping from OpenStreetMap, integration of all data into the predefined grid for a population from census and calculating an index. A technical summary of how to download and process satellite and spectral data from Sentinel-2 to obtain NDVI (Normalized Difference Vegetation Index) is described at https://rpubs.com/iv3e/ndvi. The GitHub repository includes also a dataset used in the presented analysis.
