# Supplementary material for: Mapping urban well-being with Quality Of Life Index (QOLI) at the fine-scale of grid data
Source: Sci Rep. 2024 Apr 27;14:9680. doi: 10.1038/s41598-024-60241-0 (PMC11055921; doi:10.1038/s41598-024-60241-0)
Supplement: Supplementary file 1 — Supplementary Information. [file 41598_2024_60241_MOESM1_ESM.docx]

**Appendix 1: Additional facilities in each category**

| **Category** | **Additional facilities** |
| --- | --- |
| **Dining** | Bar, pub, beer garden, food court |
| **Transport** | Boat rental, car rental, taxi |
| **Healthcare** | Hospital, veterinary, psychotherapist, social facility, nursing home, alternative, blood donation, counselling, laboratory, optometrist, physiotherapist, podiatrist, rehabilitation, sample collection, speech therapist |
| **Education** | Driving school, language school, music school, childcare, collage |
| **Sport** | Badminton, judo, tennis, athletics, gymnastics, karate, ultimate |
| **Shopping** | Shoes, clothes, health food, alcohol, butcher, coffee, chocolate, cheese, tea, baby goods, boutique, jewellery, second hand, hairdresser, tattoo, florist, hardware, furniture, antiques, electronics, sports, art, games, books, newsagent, stationery, laundry, outpost, party, pet, toys, tobacco |
| **Nature** | Hill, stone, flowerbed |

**Appendix 2: Facilities included in the category ‘other’**

| **Type** | **Facilities** |
| --- | --- |
| **Services** | charging station, vehicle inspection, car wash, carpenter, electrician, jewellery, optician, photographer, shoemaker, tailor, accountant, architect, engineer, it, lawyer, tax advisor, travel agent, tattoo, laundry, post office, hairdresser |
| **Leisure** | events venue, internet cafe, bbq, bandstand, bowling alley, sauna, stadium, cinema, casino, theme park, zoo, attraction, miniature golf, playground, nightclub, picnic site, horse riding, ice rink, adult gaming centre, beach resort, dance, dog park, amusement arcade, escape game |
| **Financial** | bank, atm, bureau de change |
| **Historic** | castle, monument, archaeological site |
| **Government** | government, diplomatic, townhall |
| **Cultural** | Museum, theatre, artwork, gallery |
| **Safety** | Shelter, police, fire station, courthouse |
| **Landscape** | fountain, nature reserve, garden, viewpoint, marina |
| **Tourism** | information, motel, hotel, guest house, hostel |
| **Other** | car sharing, conference centre, studio, drinking water, recycling, ferry terminal, give box, fuel, place of worship, planetarium, ruins, fitness centre, parcel lockers, outpost |

**Appendix 3: Distribution of non-per-capita quality of life in Warsaw**

**
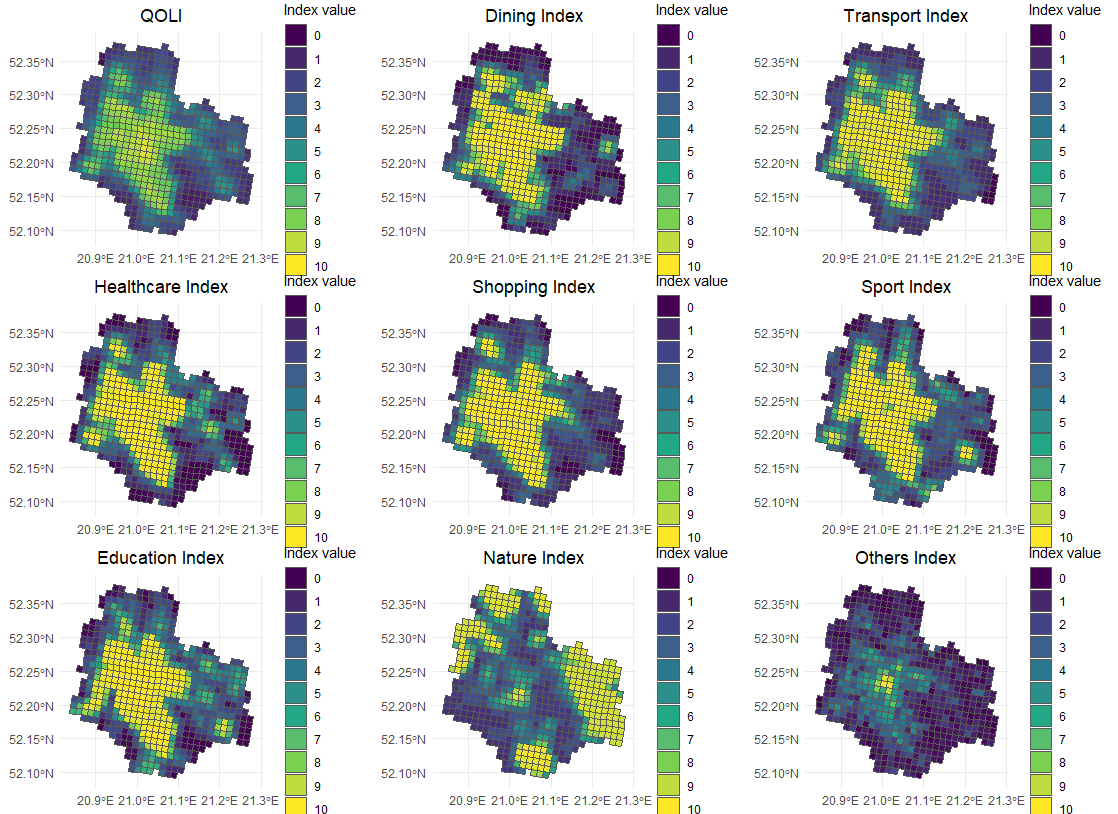
**

**Appendix 4: Internet resources for paper reproducibility**

QOLI index is fully replicable for a majority of world locations. R codes for calculations of the index are available at the GitHub repository at <https://github.com/e-dobrowolska/QOL-paper>. The R codes include data scrapping from OpenStreetMap, integration of all data into the predefined grid for a population from census and calculating an index. A technical summary of how to download and process satellite and spectral data from Sentinel-2 to obtain NDVI (Normalized Difference Vegetation Index) is described at <https://rpubs.com/iv3e/ndvi>. The GitHub repository includes also a dataset used in the presented analysis.

**Appendix 5: Testing the robustness of the index**

The design of the index involved a few decisions, which were taken after analysing the behaviour of the index.

First, we considered the types and number of categories (rows in Table 1) to include - the final decision was to include 7 named categories and one extra for 'other'. All these are amenities available in the neighbourhood, while the index does not include phenomena such as weather, crime, pollution, economic conditions (unemployment, salary), natural hazards, traffic, taxes, etc. [60]. They were not included because their spatial distribution within the city is often relatively similar and stable, while the main difference is between cities, which is beyond the scope of this index. More than 7 categories make them excessively fragmented, while less is too aggregated. The 7 categories selected (food, transport, health, education, sport, shopping, nature) divide the amenities intuitively and express well the daily needs of the inhabitants. Finally, the index is the average of the sub-indices for these 7 categories. Mathematically speaking, the more categories included, the smaller the impact of outliers. Therefore, an alternative to include only 4 categories, for example, would make the average more sensitive to outliers and would make it more difficult to classify particular amenities into categories. On the other hand, an alternative to include, say, 12 categories would make the average more smooth, but the amenities would be more fragmented.

Secondly, we considered how many levels of amenities’ importance to add to the index (columns in Table 1). This is about the hierarchy of needs and the necessity of amenities in the daily lives of residents. This is also related to the diversity of amenities, the level of socio-economic development and wealth. An extreme solution would be to have only one level (primary), which would flatten the local diversity that is often a strength of neighbourhoods. We decided to include four levels (primary, secondary, third and fourth), which is probably close to exhausting the subjective perception of individuals about the hierarchy of amenities.

Third, we considered a structure of the impact of hierarchical levels on each sub-index. This is done by assigning weights to each level. As shown in Figure 1, 90% of the impact is allocated to the primary level, while the remaining 10% is allocated to the other levels: 3% to the secondary level (one amenity), 4% to the third level (two amenities, 2% each) and 3% to the remaining amenities of the fourth level (any number). A majority weighting (90%) at the primary level means that basic amenities are valued the most, while a reduction in this weighting means that all well-equipped places are valued equally, while places with few amenities will worsen their overall ranking. Thus, the lower the primary level weight, the greater the diversity (variance) of the grid units. The strategic question remains: do people really need the second, third and fourth level amenities to live? If so, the weighting structure can be changed to promote the primary level less. If the weight of the primary level is reduced, the overall value of the index will decrease slightly - this is a natural phenomenon, as the higher level amenities may be absent and therefore their impact is zero.

This phenomenon has been illustrated with a simulation of the index shown as blue bars at the bottom of Figure A1. The first graph (Fig. A1a) is for the described index for a hypothetical grid cell (QOLI=5.76) with a primary weight of 90%. The second graph (Fig.A1b) is for the 80% weight for the primary level (scores from 0-8 instead of 0-9) and multiplying by 2 the impact of other levels (QOLI=5.4). The third graph (Fig.A1c) is for the 70% weight for the primary level (values from 0-7 instead of 0-9) and multiplying by 3 impacts of other levels (QOLI=5.03). As can be seen, the overall value of QOLI is decreasing, while the impact of the lower blue bar is decreasing. Other top bars may be missing in areas with low amenities, which increases the variance between the sub-indices and the final figures.

**Figure A1: Alternative scenarios of index design for a hypothetical grid cell: a) final index with 90% weight for primary level, b) optional design with 80% weight for primary level, c) optional design with 70% weight for primary level**

| a) |  |
| --- | --- |
| b) | **_ _ _** Index for a given cell  (*average from category sub-indices*) |
| c) |  |
